# The association between mood and anxiety disorders, and coronary heart disease in Brazil: a cross-sectional analysis on the Brazilian longitudinal study of adult health (ELSA-Brasil)

**DOI:** 10.3389/fpsyg.2015.00187

**Published:** 2015-02-25

**Authors:** Andrew H. Kemp, Andre R. Brunoni, Maria A. Nunes, Itamar S. Santos, Alessandra C. Goulart, Antonio L. Ribeiro, Isabela M. Benseñor, Paulo A. Lotufo

**Affiliations:** ^1^Center for Clinical and Epidemiologic Research, University Hospital and Faculty of Medicine, University of São Paulo, São PauloBrazil; ^2^School of Psychology and Discipline of Psychiatry, University of Sydney, Sydney, NSWAustralia; ^3^Faculty of Medicine, Federal University of Rio Grande do Sul, Porto AlegreBrazil; ^4^Hospital das Clínicas and Faculty of Medicine, Universidade Federal de Minas Gerais, Belo HorizonteBrazil

**Keywords:** major depressive disorder, anxiety disorders, disorder comorbidity, coronary heart disease

## Abstract

**Background:** Associations between major depressive disorder (MDD) and coronary heart disease (CHD) have been established, and these associations increase risk of future morbidity and mortality. Prior research has been carried out in high-income countries. Here we examine associations between the mood and anxiety disorders, and CHD in a large cohort at baseline from Brazil, a country facing a variety of challenges that may affect these associations.

**Methods:** Participants included 15,105 civil servants aged 35 to 74 at baseline (2008–2010) from the Brazilian Longitudinal Study of Adult Health (ELSA-Brasil). CHD (*N* = 721) included self-reported angina pectoris (*n* = 305), myocardial infarction (*n* = 259) and coronary revascularization (*n* = 239). Hierarchical logistic regression analyses were conducted to estimate odds ratios and confidence intervals.

**Results:** Major findings indicate that comorbid MDD and anxiety disorders (*n* = 434) are associated with a threefold increase in CHD, MDD alone (*n* = 170) with a twofold increase in CHD, while generalized anxiety disorder alone (*n* = 1,394) and mixed anxiety and depression disorder (*n* = 1,844) – symptoms present, but diagnostic threshold not reached – are associated with a 1.5-fold increase in CHD, after full adjustment for covariates.

**Conclusion:** The association with CHD is greatest in those with psychiatric comorbidity, while associations were also observed in MDD and generalized anxiety disorder without comorbidity. While findings are limited by the cross-sectional design of the study, given the known risks associated with comorbidity of the mood and anxiety disorders with CHD, findings reinforce the importance of comprehensive health assessment in Brazil.

## INTRODUCTION

Major depressive disorder (MDD) and coronary heart disease (CHD) are leading burdens of disease and the relationship between these disorders is bidirectional (Nemeroff and Goldschmidt-Clermont, 2012; Ramasubbu et al., 2012a; Stapelberg et al., 2012; Lichtman et al., 2014): many patients experience depression decades before CHD is manifest, while MDD in patients with CHD is associated with increased morbidity and mortality. However, it remains unclear whether MDD, anxiety disorder or their comorbidity are most associated with CHD. Research has begun to highlight associations between the anxiety disorders and CHD (Tully and Cosh, 2013; Tully et al., 2013). A study on participants recruited for the Netherlands Study of Depression and Anxiety (*N* = 2807) reported that persons with current anxiety disorders with or without depression have a threefold increased prevalence of CHD, while no associations were observed for those with depressive disorders alone, or for depressive and anxiety disorders in remission (Vogelzangs et al., 2010). Research has been restricted to high-income countries including Australia, Canada, Denmark, Finland, Netherlands, UK, and USA (Vogelzangs et al., 2010; Tully and Cosh, 2013), highlighting a need for research in low to middle-income countries. Given the lack of data on the associations between common mental disorders (CMD), specific mood and anxiety disorders, and CHD in Brazil, an upper-middle-income country, the present study sought to address this need.

It is unclear whether the associations between the mood and anxiety disorders, and CHD parallel those or differ from the findings reported in developed countries such as the Netherlands (e.g., Vogelzangs et al., 2010). Research has demonstrated that Brazil is characterized by higher rates of mental disorders (Andrade et al., 2012), while rates of antidepressant use (Brunoni et al., 2013) are lower than high-income countries. Brazil faces significant social challenges that may contribute to psychological distress (de Jesus Mari, 2014), which may affect observed associations. For example, Brazil has one of the highest levels of income inequality in the world and recent data from this country (Filho et al., 2013) indicate that this inequality is associated with mental disorders, especially depression. The authors argued that inequality is associated with adverse social comparisons, leading to psychological distress, and increases in disease and mortality, consistent with the ‘relative income hypothesis’ (Wilkinson, 2002). Income inequality is likely one of many psychosocial stressors that may affect the associations between the mood and anxiety disorders and CHD in Brazil. We hypothesized a relationship between CMD and CHD, and predicted that the prevalence of CHD would be most robust in those with comorbid MDD and anxiety disorders after controlling for relevant confounding factors including antidepressant medications, socio-demographic issues, physical inactivity, obesity and smoking, as well as risk factors including dyslipidemia, hypertension, and diabetes. Here we defined CHD as participants reporting a medical history of stable angina pectoris, myocardial infarction (MI) or coronary revascularisation.

## MATERIALS AND METHODS

### PARTICIPANTS

ELSA-Brasil is a cohort of 15,105 civil servants aged 35 to 74 enrolled between August, 2008 and December, 2010 at six cities (Belo Horizonte, Porto Alegre, Rio de Janeiro, Salvador, Sao Paulo, and Vitoria) designed to investigate the relationship between cardiovascular diseases and diabetes, their social determinants and risk factors. The study design and sampling procedures of ELSA-Brasil have been reported previously (Aquino et al., 2012; Schmidt et al., 2014). Exclusion criteria comprised current or recent pregnancy (within 4 months of first interview), intention to quit working at the institution in the near future, severe cognitive or communication impairment, and if retired, residence outside of a study centre’s metropolitan area.

### ETHICS STATEMENT

The ethics committees of the participating universities approved the research protocol. All participants provided written informed consent after a complete description of the study. The study design and sampling procedures of ELSA-Brasil have been reported previously (Aquino et al., 2012; Schmidt et al., 2014).

### PSYCHIATRIC EVALUATION

Mental disorders were determined by trained interviewers using the Portuguese version (Nunes et al., 2012) of the Clinical Interview Schedule-Revised (CIS-R; Lewis et al., 1992). This is a structured interview used for diagnosis of current, common, non-psychotic psychiatric conditions in the community. The complete CIS-R version was applied; CMDs were defined as participants with CIS-R scores ≥12. The following ICD-10 categories were also determined: MDD, agoraphobia, social phobia, specific phobia, generalized anxiety disorder (GAD), panic disorder, social anxiety disorder, and mixed anxiety and depressive disorder (MADD). The ICD-10 refers to the 10th revision of the International Statistical Classification of Diseases and Related Health Problems, a medical classification list by the World Health Organization. These diagnostic groupings were then reduced to six categories: MDD, GAD, all phobias, panic disorder, a comorbid group comprised of participants with MDD and any anxiety disorder, and MADD. The latter two categories are distinct; the former comprises participants that meet criteria for both MDD and any anxiety disorder, while the latter comprises participants who display symptoms of both anxiety and depression, but do not meet the criteria of either diagnosis separately.

### CORONARY HEART DISEASE ASSESSMENT

Coronary heart disease included participants who reported a medical diagnosis of stable angina pectoris, MI or coronary revascularization determined through questionnaire- and intensive interview-based assessment focusing on medical history.

### COVARIATES

Sociodemographic information included age, sex, years of education (less than high-school, high-school, university) and race (white, mixed, black, Asiatic, indigenous). Lifestyle and behavioral characteristics included smoking status (never, past/current), physical activity (measured with the International Physical Activity Questionnaire (Craig et al., 2003) and categorized according to low, moderate, or high activity based on scoring guidelines: http://www.ipaq.ki.se/scoring.pdf) and body mass index (BMI; weight in kilograms divided by height in meters squared). Established risk factors for CHD were also considered in analysis; these included hypertension, diabetes mellitus (DM), and dyslipidemia. Hypertension was defined as systolic blood pressure ≥140 mm Hg, or diastolic blood pressure ≥90 mm Hg, or use of antihypertensive medications. Resting blood pressure was measured three times in the seated position after 5 min rest, and the average of the second and third measurements were used in analyses. Diabetes was defined as self-reported or fasting blood glucose ≥126 mg/dL or 2-h oral glucose tolerance test ≥200 mg/dL or glycated hemoglobin ≥6.5%. Dyslipidemia was defined as LDL-cholesterol ≥130 mg/dL or use of lipid reductor. We identified antihypertensive treatments, medications for diabetes, and antidepressants on the basis of prescription and over-the-counter drugs in the past 2 weeks determined through pill bottle review. Antidepressant use was added as a covariate considering that antidepressant medications may also contribute to morbidity and mortality (Smoller et al., 2009; Whang et al., 2009; Hamer et al., 2011).

### STATISTICAL ANALYSIS

Statistical analysis was conducted using IBM SPSS Statistics Version 21. Participant characteristics were examined using independent samples *t*-tests and one-way analyses of variance (ANOVA) for contrasts involving continuous dependent measures, and χ^2^ statistics for categorical variables. Tukey’s HSD was used to correct for multiple comparisons and aid interpretation of ANOVA’s, while standardized residuals (*z*-scores) were used to help interpret chi-square tests on larger contingency tables (as per Field, 2013). A series of logistic regression analyses were then used to estimate the odds ratios and 95% confidence intervals for the association between CMD and CHD, before and after adjustment for covariates. These initial analyses were followed up by sensitivity analysis on the specific mental disorder groupings. Unadjusted analyses (Model 1) were conducted on CMD, as well as specific mental disorder groupings, with no other predictors. Adjusted multivariate analyses (Model 2) involved hierarchical (blockwise) logistic regression analysis on *a priori* selected covariates (as described in previous section). Each covariate grouping (i.e., sociodemographic information, lifestyle/behavioral characteristics, risk factors, and medications) was entered into a successive block; mental disorders were entered into the final block. Variables in each block were entered simultaneously using the forced entry method to assess the independent contribution of each predictor over and above all other predictors entered in the same block. Finally, additional sensitivity analyses were conducted for subtypes of CHD including stable angina pectoris, MI, and coronary revascularisation to determine the consistency of associations across these distinct categories of CHD. Again, these analyses involved hierarchical (blockwise) logistic regression analysis adjusting for covariate groupings (as per Model 2).

## RESULTS

### PARTICIPANT CHARACTERISTICS

**Table 1** summarizes participant characteristics by CHD status. Compared to participants without CHD (*n* = 14,366), those with CHD (*n* = 721) were older, black, less educated, and male. They engaged in less physical activity, were heavier, more likely to have smoked (or to be a smoker), have DM, hypertension, dyslipidemia, or a CMD and to be medicated with antidepressants. When groupings of mental disorders were examined, comorbid MDD and anxiety was significantly associated with CHD. **Table 2** presents participant characteristics according to whether or not participants were identified as having a CMD. Compared to those without CMD (*n* = 11,058), participants with CMD (*n* = 4,036) were more likely to be younger, less educated, and female. They were less likely to be white, engaged in less physical activity, and were heavier. They were more likely to have smoked (or to be a smoker), to have DM or CHD, and to be medicated with antidepressants. **Table 3** provides finer grained detail on participant characteristics by specific mental disorder groupings including MDD (*n* = 179), GAD (*n* = 1,458), phobias (*n* = 156), PD (*n* = 74), MADD (*n* = 1914), comorbid MDD, and anxiety disorders (*n* = 451) as well as controls.

**Table 1:** Participant characteristics by coronary heart disease (CHD) status^1^.

Characteristics	No CHD (*n* = 14366, 93%)	CHD (*n* = 721, 7%)	Statistic
Age, mean (SD)	51.75 (8.97)	58.71 (8.75)	*t*(15085) = 20.34, *p* < 0.001
Women (%)	54.9	45.5	χ^2^(1) = 24.28, *p* < 0.001
Education (%)			χ^2^(2) = 85.03, *p* < 0.001
Less than high-school High-school College	12.1 34.7 53.2	23.6 33.6 42.9
Race (%)			χ^2^(4) = 7.76, *p* = 0.101
White Mixed Black Asiatic Indigenous	52.4 28.2 15.9 2.5 1.0	50.1 27.1 19.2 2.1 1.6
Smoker (past or current) (%)	42.5	55.9	χ^2^(1) = 50.39, *p* < 0.001
Physical activity			χ^2^(2) = 8.59, *p* = 0.014
Low Moderate High	76.9 13.9 9.2	77.7 16.0 6.3
Body mass index (kg/m^2^), mean (SD)	26.95 (4.74)	28.40 (4.82)	*t*(15079) = 7.99, *p* < 0.001
Antidepressant status (yes) (%)	6.0	8.7	χ^2^(1) = 8.39, *p* = 0.004
Diabetes mellitus (DM; yes) (%)	18.8	37.7	χ^2^(1) = 156.38, *p* < 0.001
Hypertension (yes) (%)	34.0	70.0	χ^2^(1) = 384.40, *p* < 0.001
Dyslipidemia (yes) (%)	56.9	75.9	χ^2^(1) = 101.50, *p* < 0.001
Mental disorder status (%)			χ^2^(1) = 28.04, *p* < 0.001
No mental disorder Common mental disorder (CMD)	73.7 26.3	64.8 35.2
Mental disorder groupings^2^ (%)			χ^2^(1) = 20.05, *p* = 0.003
MDD GAD Phobias PDMADD Comorbid	1.2 9.9 1.0 0.5 13.0 2.9	2.0 10.2 1.5 0.7 14.6 6.6

*^1^Some proportions might not add up to due to rounding, and small numbers of participants with missing data. ^2^Participants categorized into major depressive disorder (MDD), generalized anxiety disorder (GAD), and panic disorder (PD) did not have additional comorbidity. The Phobias category includes Agoraphobia with or without panic disorder, Social Phobias and Specific Phobias. mixed anxiety and depressive disorder (MADD) includes participants who display symptoms of both anxiety and depression, but do not meet the criteria of either diagnosis separately, while the Comorbid group (comorbid anxiety and depression) includes participants that meet criteria for MDD and any anxiety disorder.*

**Table 2:** Participant characteristics by CMD status^1^.

Characteristics	No CMD (*n* = 11058, 73%)	CMD (*n* = 4036, 27%)	*p*-value
Age, mean (SD)	52.60 (9.22)	50.70 (8.53)	*t*(7703.15) = 11.85, *p* < 0.001
Women (%)	49.2	68.7	(1) = 451.06, *p* < 0.001
Education (%)			(2) = 160.27, *p* < 0.001
Less than high-school High-school College	12.3 32.0 55.7	14.0 41.7 44.3
Race (%)			(4) = 133.98, *p* < 0.001
White Mixed Black Asiatic Indigenous	54.7 26.6 15.0 2.8 0.9	45.3 32.4 19.1 1.8 1.4
Smoker (past or current) (%)	42.3	45.2	(1) = 10.26, *p* < 0.001
Physical activity			(2) = 164.39, *p* < 0.001
Low Moderate High	74.4 15.3 10.4	84.1 10.3 5.5
Body mass index (kg/m^2^), mean (SD)	26.84 (4.61)	27.58 (5.08)	*t*(6602.94) = 8.31, *p* < 0.001
Antidepressant status (yes) (%)	4.6	10.5	(1) = 180.53, *p* < 0.001
DM (yes) (%)	19.1	21.2	(1) = 8.078, *p* = 0.004
Hypertension (yes) (%)	35.7	36.0	(1) = 0.082, p = 0.775
Dyslipidemia (yes) (%)	58.3	56.4	(1) = 4.64, *p* = 0.031
CHD status (%)			(1) = 28.04, *p* < 0.001
No Yes	95.8 4.2	93.7 6.3

^1^Some proportions might not add up to 100 due to rounding, and small numbers of participants with missing data.

**Table 3:** Participant characteristics by mental disorder groupings^1,2^.

Characteristics	CTL (*n* = 10412)	MDD (*n* = 179)	Phobias (*n* = 156)	PD (*n* = 74)	GAD (*n* = 1458)	MADD (*n* = 1914)	Comorbid (*n* = 451)	*p*-value
Age, mean (SD)	52.66 (9.23)	50.87 (8.99)	50.15* (8.40)	52.69 (8.48)	50.92* (8.50)	50.72* (8.71)	51.25* (8.44)	*F*(6,14650) = 20.58, *p* < 0.001
Women (%)	48.4*	74.9*	53.5	66.2	69.6*	66.4*	75.1*	χ^2^(6) = 508.12, *p* < 0.001
Education (%)								χ^2^(12) = 165.07, *p* < 0.001
Less than high-school High-school College	12.4 31.8* 55.8*	14.0 37.4 48.6	17.8 45.9* 36.3*	24.3* 44.6 31.1*	12.3 39.4* 48.3*	12.7 38.8* 48.6*	16.3* 48.5* 35.2*
Race (%)								χ^2^(24) = 160.13, *p* < 0.001
White Mixed Black Asiatic Indigenous	54.8* 26.6* 14.9* 2.7 0.9	55.1 27.0 14.6 2.8 0.6	43.5 33.1 22.7* 0* 0.6	25.7* 36.5 33.8* 2.7 1.4	49.9 29.5 16.7 2.6 1.3	44.5* 32.9* 19.6* 1.9 1.2	41.6* 35.4* 19.2 1.5 2.2*
Smoker (past or current), %	42.2	50.8	45.2	52.7	44.4	42.9	49.6*	χ^2^(12) = 19.35, *p* = 0.004
Physical activity								χ^2^(12) = 149.18, *p* < 0.001
Low Moderate High	74.3* 15.3* 10.4*	85.1 9.1 5.7	85.3 9.6 5.1	78.1 15.1 6.8	80.1 12.6 7.3*	83.8* 10.3* 6.0*	88.6* 7.6* 3.8*
BMI (kg/m^2^), mean (SD)	26.81 (4.58)	27.90* (5.11)	27.71 (5.34)	27.01 (4.71)	27.36* (5.03)	27.48* (5.03)	27.93* (5.32)	*F*(6,14644) = 12.17, *p* < 0.001
Hypertension (yes) (%)	35.7	34.6	38.9	47.3	36.6	35.0	36.6	χ^2^(6) = 6.07, *p* = 0.42
Diabetes (yes) (%)	19.0	22.9	24.2	29.7*	18.8	21.3	22.9	χ2(6) = 17.57, *p* = 0.007
Dyslipidemia (yes) (%)	58.5	53.7	53.8	60.8	57.2	56.4	55.8	χ^2^(6) = 6.89, *p* = 0.331
Antidepressant Use (yes) (%)	4.4*	13.6*	8.9	9.5	10.9*	7.3*	15.8*	χ^2^(6) = 217.86, *p* < 0.001
CHD (yes) (%)	4.3*	7.8	6.4	6.8	4.8	5.2	10.0*	χ^2^(6) = 39.47, *p* < 0.001

^1^Some proportions might not add up to 100% due to rounding, and small numbers of participants with missing data. *Refers to one-way ANOVA in which disorder is compared to controls (Tukey’s HSD, *p* < 0.05) or standardized residuals (*z*-scores) from χ^2^ statistics lying outside ±1.96 reflecting a significance of value of *p* < 0.05.

### ASSOCIATION BETWEEN MENTAL DISORDERS AND CHD

**Table 4** describes the results of unadjusted and fully adjusted logistic regression analyses assessing the association of CMD as well as mental disorder groupings relative to those without mental disorders. Model 1 provides the results of unadjusted analyses conducted for CMD [model χ^2^(1) = 26.53, *p* = 0.001] and specific mental disorder groupings [model χ^2^(6) = 32.16, *p* < 0.001]. Model 2 refers to adjusted analyses for CMD [model χ^2^ (17) = 708.25, *p* < 0.001; block χ^2^ (1) = 43.76, *p* < 0.001] and mental disorder groupings [model χ^2^ (22) = 686.87, *p* < 0.001; block χ^2^ (6) = 43.06, *p* < 0.001]. In the fully adjusted model (model 2), the odds of CHD were most robust for those with comorbid MDD and anxiety disorders (OR: 2.99, 95% CI = 2.10–4.28), followed by those participants with MDD (OR: 2.14, 95% CI = 1.17–3.95), participants with GAD (OR: 1.41, 95% CI = 1.07–1.85), and participants that displayed symptoms of both anxiety and depression, but did not meet the criteria of either diagnosis separately (MADD; OR: 1.53, 95% CI = 1.20–1.94). Participants with comorbid MDD and anxiety disorders display a greater association with CHD, than GAD and MADD (i.e., confidence intervals do not overlap), but not MDD.

**Table 4:** Unadjusted (model 1) and adjusted (model 2) associations of mental disorders with CHD.

	CHD: model 1^a^ (*n* = 721)				CHD: model 2^b^ (*n* = 721)			
Predictor	*N*	OR	95% CI	*p*-value	*N*	OR	95% CI	*p*-value
**Common mental disorder (CMD)**
No CMD	11050		REF		10631		REF	
CMD	4026	1.526	1.304–1.787	<0.001	3869	1.829	1.535–2.179	<0.001
**Mental disorder groupings**
No mental disorder	10405		REF		10012		REF	
MDD	179	1.908	1.097–3.320	0.022	170	2.144	1.165–3.945	0.014
Phobias	156	1.540	0.806–2.944	0.191	151	1.555	0.763–3.169	0.224
PD	74	1.630	0.654–4.059	0.845	73	1.433	0.553–3.708	0.459
GAD	1457	1.135	0.877–1.469	0.337	1394	1.405	1.068–1.849	0.015
MADD	1914	1.240	0.992–1.549	0.059	1844	1.528	1.203–1.941	0.001
Comorbid	451	2.492	1.806–3.440	<0.001	434	2.994	2.096–4.275	<0.001

^a^Model 1 relates to separate unadjusted analyses for CMD and specific mental disorder groupings including MDD, Phobias, panic disorder (PD), generalized anxiety disorder (GAD), mixed anxiety and depressive disorder (MADD), and Comorbid MDD and any anxiety disorder. ^b^Model 2 relates to analyses adjusted for sociodemographic variables (age, sex, years of education, ethnicity), lifestyle and behavioral characteristics (smoking status, physical activity, body mass index), risk factors (hypertension, diabetes, dyslipidemia), and medications for lipid reduction, hypertension, diabetes, and mental disorders (antidepressants).

### ASSOCIATION BETWEEN MENTAL DISORDERS AND CHD SUBTYPES

**Table 5** reports results for additional sensitivity analyses on stable angina pectoris, MI, and coronary revascularization.

**Table 5:** Association between mental disorders and CHD subtypes fully adjusted for sociodemographic variables, lifestyle and behavioral characteristics, risk factors, and medications.

	Angina (*n* = 305)				Myocardial infarction (MI) (*n* = 259)				Coronary revascularisation (*n* = 239)			
Predictor	*N*	OR	95% CI	*p*-value	*N*	OR	95% CI	*p*-value	*N*	OR	95% CI	*p*-value
**Common mental disorder (CMD)**
No CMD	10358		REF		10634			REF	10635			REF
CMD	3755	2.134	1.671–2.724	<0.001	3877	1.615	1.213–2.151	0.001	3878	1.431	1.048–1.954	0.024
**Mental disorder groupings**
No mental disorder	9754		REF		10014			REF	10015		REF	
MDD	165	2.866	1.359–6.043	0.006	170	2.142	0.818–5.611	0.121	170	0.427	0.057–3.215	0.408
Phobias	146	1.616	0.585–4.469	0.355	152	2.191	0.843–5.696	0.107	152	0.470	0.063–3.490	0.461
PD	71	2.117	0.648–6.914	0.214	73	0.615	0.081–4.653	0.637	73	1.691	0.381–7.503	0.489
GAD	1362	1.588	1.092–2.310	0.015	1395	1.219	0.759–1.957	0.413	1395	1.330	0.818–2.162	0.250
MADD	1795	1.639	1.175–2.286	0.004	1847	1.536	1.045–2.255	0.029	1847	1.447	0.951–2.201	0.084
Comorbid	412	2.976	1.831–4.834	<0.001	436	3.051	1.743–5.342	>0.001	436	3.087	1.668–5.713	<0.001

For angina, there was a significant association with CMD [model χ^2^(17) = 199.51, *p* < 0.001; block χ^2^(1) = 35.49, *p* < 0.001], and mental disorder groupings [model χ^2^(22) = 189.43, *p* < 0.001; block χ^2^(6) = 27.57, *p* < 0.001] after full adjustment. Results indicate that MDD, GAD, MADD, and comorbid MDD and anxiety disorders are associated with angina; odds were most robust for the comorbid group (OR: 2.98, 95% CI = 1.83–4.83), followed by MDD (OR: 2.87, 95% CI = 1.36–6.04), GAD (OR: 1.58, 95% CI = 1.09–2.31), and MADD (OR: 1.64, 95% CI = 1.18–2.29). It should be noted that confidence intervals are overlapping for each of these groups, so we cannot say with certainty that one group displays a ‘greater’ association with CHD over another.

For MI, there was a significant association with CMD [model χ^2^(17) = 447.07, *p* < 0.001; block χ^2^(1) = 10.32, *p* < 0.001), and mental disorder groupings [model χ^2^(22) = 442.72, *p* < 0.001; block χ^2^(6) = 18.41, *p* = 0.005] after full adjustment. Results indicate that MADD and Comorbid groupings are associated with MI; odds were most robust for the Comorbid group (OR: 3.051, 95% CI = 1.74–5.34) followed by the MADD group (OR: 1.54, 95% CI = 1.05–2.26). Again, confidence intervals are overlapping for Comorbid and MADD groupings, thus we cannot say that the Comorbid group displays a ‘greater’ association with MI, than MADD. For coronary revascularisation, there was a significant association with CMD [model χ^2^(17) = 483.15, *p* < 0.001; block χ^2^(1) = 4.88, *p* = 0.027], and mental disorder groupings [model χ^2^(22) = 479.79, *p* < 0.001; block χ^2^(6) = 15.01, *p* = 0.02] after full adjustment. Results indicate that the Comorbid group only is associated with coronary revascularisation (OR: 3.087, 95% CI = 1.67–5.71)

## DISCUSSION

The goal of this study was to determine the associations between CMD, specific mood and anxiety disorder groupings, and CHD in a large cohort of participants recruited for the ELSA-Brasil project. Major findings highlight a significant association between CMD and CHD (OR: 1.83, 95% CI = 1.53–2.18), even after full adjustment for sociodemographic variables, lifestyle and behavioral characteristics, and additional risk factors including dyslipidemia, hypertension, diabetes, and medication use. Further analysis revealed that this association is most apparent in MDD, GAD, MADD and comorbid MDD, and anxiety. No significant associations were observed for phobias or PD. The association was most robust for those with comorbid MDD and anxiety (OR: 2.99, 95% CI = 2.10–4.28), followed by MDD (OR: 2.14, 95% CI = 1.17–3.95), MADD (OR: 1.53, 95% CI = 1.1.20–1.94), and GAD (OR: 1.41, 95% CI = 1.07–1.85). Notably, participants with comorbid MDD and anxiety disorders display a greater association with CHD, than GAD and MADD, but not MDD, a specific finding that we discuss further below. Examination of CHD subtypes also revealed important information; while MDD, GAD, MADD and comorbid MDD, and anxiety were associated with a significant increase in odds for angina, only those with comorbid MDD and anxiety disorders and MADD displayed an increase in odds for MI and coronary revascularisation. In summary, findings highlight (1) strong associations between psychiatric comorbidity and CHD in Brazil, (2) associations for MDD and GAD, in addition to MADD and the comorbid group, with angina (without a history of MI or revascularisation), emphasizing the need for close monitoring of these individuals to avoid further adverse events, and (3) that psychiatric comorbidity is associated with MI and coronary revascularization.

The most robust associations with CHD were observed in those individuals with psychiatric comorbidity. It is worth noting here that the combined presence of anxious and depressive symptoms is associated with more than a twofold increased risk of mortality in patients with ischemic heart disease (Doering et al., 2010), relative to those who were free from psychiatric symptoms. We have previously shown that reduced heart rate variability – a predictor of future mortality – is most reduced in MDD patients with GAD (Kemp et al., 2012), demonstrating the adverse effects of distinctive clinical features including a depressive episode, persistent worry, and hypervigilance on cardiac function (see also Kemp et al., 2014). In the present study, we also observed that only disorders with concurrent anxiety and depression (comorbid MDD and anxiety, and MADD) also displayed associations with ‘hard’ CHD endpoints including MI and coronary revascularisation. While our findings are consistent with studies from high-income countries (e.g., Vogelzangs et al., 2010), highlighting greater associations with comorbid anxiety and depression, our study also highlights associations with CHD in participants without psychiatric comorbidity. In particular, we observed associations between MDD and GAD, and CHD, further highlighting the importance of non-comorbid, chronic depression, and generalized anxiety, rather than disorders involving heightened phasic anxiety (the phobias and panic disorder). These findings further suggest that characteristic features of depression (anhedonia), generalized anxiety (anxious apprehension), and psychiatric comorbidity (trait negative affect) may all contribute to the associations we observed here in Brazil.

Prior research has indicated that Brazil is a country where depression (and presumably anxiety) is most often somaticized (Simon et al., 1999; see also Mari et al., 2013). While this suggests that the associations we observed here may in part, reflect an overlap of somatic symptoms observed in mental illness and physical disease, the importance of this finding should not be minimized. Prognosis for patients with angina is not benign; in fact, it is no better than those with MI and/or revascularization (e.g., Buckley and Murphy, 2009). Some limitations of this study are worth noting here. These include the cross-sectional design of the study, generalizability of the sample beyond civil servants in Brazil, and the possibility of undetected or undiagnosed CHD considering that classification of CHD was based on self-report. The cross-sectional design of the study makes it impossible to determine whether the mood and anxiety disorders preceded CHD or vice versa. That said, the literature suggests that the relationship between MDD (and anxiety) and CHD is bidirectional and reciprocal (Nemeroff and Goldschmidt-Clermont, 2012; Ramasubbu et al., 2012a; Stapelberg et al., 2012). We also note that the ELSA-Brasil cohort captures the racial, social, and regional diversity in the Brazilian population (see Schmidt et al., 2014), highlighting the relevance of the present study’s findings for developing countries. It is possible, however, that non-diagnosed CHD is *lower in our sample*, than for the general Brazilian population, as the civil servants who participated in our study have more access to health services. It is possible therefore that the associations we observe here are lower than those that might be observed in the general Brazilian population. Indeed, it has been noted previously that there is a low identification rate for depression and other CMDs in Brazil (Mari et al., 2013; see also Mari et al., 1987; Busnello et al., 1999).

There are a number of potential explanations for the associations we observed between the CMD (and specific diagnostic groupings) and CHD including psychosocial and biological factors. Brazil currently faces major social challenges, which may contribute to chronic stress and increased morbidity (de Jesus Mari, 2014), strengthening the associations between mental disorders and CHD we observed here, in comparison to high-income countries. In addition to income inequality (Filho et al., 2013), personal safety remains an issue of great concern. For example, findings from a representative sample of 15 to 75-year old residents of São Paulo and Rio de Janeiro in Brazil indicated that psychiatric disorders and traumatic events, especially violence, are extremely common (Ribeiro et al., 2013). Nearly 90% of participants surveyed in that study faced a lifetime exposure to actual or threatened death, and these traumatic events correlated with all psychiatric diagnoses. These results are particularly relevant in light of other research (Russ et al., 2012) highlighting a dose-response association between psychological distress and increased risk of mortality from a variety of causes including CVD, cancer and external causes over 8 years. Biological factors may also underpin associations between the mood and anxiety disorders and CHD including autonomic dysfunction, a hyperactive hypothalamic-pituitary-adrenal axis, increased activity in the sympatho-adreno-medullary activity, increased inflammation, and platelet activation and aggregation (Nemeroff and Goldschmidt-Clermont, 2012; Ramasubbu et al., 2012a; Stapelberg et al., 2012; Lichtman et al., 2014). Decreases in heart rate variability indicative of autonomic dysfunction may provide a structural link between emotional disturbance and failure to appropriately regulate inflammatory processes leading to increased morbidity and mortality from a host of disorders including CVD’s (Kemp and Quintana, 2013). Disturbances in the immune system may also lead to depression (Dantzer et al., 2008), highlighting the bidirectional relationships between the mental disorders, and physical disease.

Our study is characterized by a number of strengths including a focus on a relatively large and well-characterized sample of the Brazilian population, consideration of a variety of specific mood and anxiety disorder groupings, application of a structured clinical interview – rather than self-report – to determine psychiatric diagnoses, and adjustment for a host of covariates known to contribute to metabolic and cardiovascular risk. Recently, we reported that that use of tricyclic antidepressants is associated with a twofold increase in prevalent CHD, above and beyond severity of mood and anxiety symptoms (Kemp et al., 2015). Importantly, we demonstrate here that the association between mood and anxiety disorders, and CHD is above and beyond use of antidepressant medications including tricyclic antidepressants. A recent statement by the Canadian Network for Mood and Anxiety Treatments (CANMAT) task force (Ramasubbu et al., 2012b) highlights the bidirectional relationship between the mood and anxiety disorders, and a variety of medical conditions including cardiovascular disease, cerebrovascular disease, cancer, human immuno-deficiency virus, hepatitis C virus, migraine, multiple sclerosis, epilepsy, and osteoporosis. Given the increased risks for morbidity and mortality associated with comorbid psychiatric and medical conditions, future studies are needed to better understand the pathways underpinning their comorbidity, with a goal toward developing more effective preventive strategies and treatments. Interested readers are referred to recent guidelines for pharmacological and psychosocial management in low and middle-income countries (Mari et al., 2013). In conclusion, the present study provides important new information on the association between the mental disorders and CHD in Brazil. Findings indicate that the most robust association with CHD relates to symptomatic and diagnostic, psychiatric comorbidity, although an association with MDD and GAD was also observed. Both MDD and GAD were found to be associated with an increase in the odds for CHD overall as well as angina pectoris, which may have adverse implications for the future health of ELSA-Brasil participants. Considering that comorbid psychiatric illness and CHD is associated with increased morbidity and mortality (Lichtman et al., 2014), findings reinforce a need for, and the importance of, comprehensive health assessment in Brazil, a country that faces major social challenges and inequities.

## AUTHOR CONTRIBUTIONS

AK conducted the literature search, identified the research questions, and clarified the hypotheses. He analyzed the data, interpreted the results and wrote the paper. MN adapted the Clinical Interview Schedule for use in our project and was responsible for the psychiatric evaluations of participants recruited in ELSA-Brasil. She also helped to refine the research questions and finalize the article for publication. AB assisted AK with reviewing the literature, and clarifying research questions and hypotheses. He played a key role in interpreting the results and writing the paper. IS and AG were involved in data collection, data analysis, interpretation of results, and writing the manuscript. AR was also involved in data collection and helped to critically revise the manuscript for publication. IB and PL have been involved in the ELSA-Brasil project since its inception, and secured the funding to initiate and conduct the project. They were involved in all aspects of the present study including research design, data collection, analysis, interpretation, and writing the manuscript.

## Conflict of Interest Statement

The authors declare that the research was conducted in the absence of any commercial or financial relationships that could be construed as a potential conflict of interest. The Review Editor Mario F. Juruena declares that, despite being affiliated to the same institution as the author Andrew Kemp, the review process was handled objectively.
